# A Stimulated Raman Scattering CMOS Pixel Using a High-Speed Charge Modulator and Lock-in Amplifier

**DOI:** 10.3390/s16040532

**Published:** 2016-04-13

**Authors:** De Xing Lioe, Kamel Mars, Shoji Kawahito, Keita Yasutomi, Keiichiro Kagawa, Takahiro Yamada, Mamoru Hashimoto

**Affiliations:** 1 Research Institute of Electronics, Shizuoka University, 3-5-1 Johoku, Nakaku, Hamamatsu, Shizuoka 432-8011,Japan; lioe@idl.rie.shizuoka.ac.jp (D.X.L.); kamel@idl.rie.shizuoka.ac.jp (K.M.); kyasu@idl.rie.shizuoka.ac.jp (K.Y.); kagawa@idl.rie.shizuoka.ac.jp (K.K.); 2 Graduate School of Engineering Science, Osaka University, 1-3 Machikaneyama, Toyonaka, Osaka 560-8531, Japan; yamada@sml.me.es.osaka-u.ac.jp (T.Y.); mamoru@me.es.osaka-u.ac.jp (M.H.)

**Keywords:** stimulated Raman scattering, CMOS image sensor, lock-in amplifier, low frequency noise, double modulation, Raman shift

## Abstract

A complementary metal-oxide semiconductor (CMOS) lock-in pixel to observe stimulated Raman scattering (SRS) using a high speed lateral electric field modulator (LEFM) for photo-generated charges and in-pixel readout circuits is presented. An effective SRS signal generated after the SRS process is very small and needs to be extracted from an extremely large offset due to a probing laser signal. In order to suppress the offset components while amplifying high-frequency modulated small SRS signal components, the lock-in pixel uses a high-speed LEFM for demodulating the SRS signal, resistor-capacitor low-pass filter (RC-LPF) and switched-capacitor (SC) integrator with a fully CMOS differential amplifier. AC (modulated) components remained in the RC-LPF outputs are eliminated by the phase-adjusted sampling with the SC integrator and the demodulated DC (unmodulated) components due to the SRS signal are integrated over many samples in the SC integrator. In order to suppress further the residual offset and the low frequency noise (1/f noise) components, a double modulation technique is introduced in the SRS signal measurements, where the phase of high-frequency modulated laser beam before irradiation of a specimen is modulated at an intermediate frequency and the demodulation is done at the lock-in pixel output. A prototype chip for characterizing the SRS lock-in pixel is implemented and a successful operation is demonstrated. The reduction effects of residual offset and 1/f noise components are confirmed by the measurements. A ratio of the detected small SRS to offset a signal of less than 10*^−^*^5^ is experimentally demonstrated, and the SRS spectrum of a Benzonitrile sample is successfully observed.

## 1. Introduction

Developments in optical imaging techniques enable better understanding of the microscopic world. One of the modalities, Raman scattering, has long been explored to provide a contrast mechanism for label-free, noninvasive imaging to study biological samples by detecting specific vibrational spectra of chemical bonds [1,2]. Spontaneous Raman scattering microscopy possesses the relatively high spatial resolution capability. However, due to the extremely small Raman cross section, the spontaneous Raman scattering microscopy requires a lengthy acquisition time and thus is not suitable for live imaging [3]. Coherent Raman scattering (CRS) microscopy enhances the Raman response significantly, which facilitates high-speed imaging. The CRS utilizes two laser sources, pump beam at frequency ω_p_ and Stokes beam at frequency ω_s_, to coincide on the sample. When the frequency difference matches a specific molecular vibrational frequency, interaction between two laser beams and molecular vibration occurs in the focal volume. There are two CRS techniques: coherent anti-Stokes Raman scattering (CARS) and stimulated Raman scattering (SRS). The CARS produces anti-Stokes emission at ω*_as_* = 2ω*_p_* − ω*_s_* by virtue of a wave-mixing process. However, CARS suffers from non-resonant background signal, which interferes with the resonant signal and consequently causes the CARS spectrum to be distorted [4,5]. A number of approaches have been carried out to extract only the quantitative data out of CARS [6]. Nonetheless, these complicate the experimental system and analysis. 

In contrast to the CARS, the SRS signal is stimulated only when the frequency difference, Ω = *ω_p_* − *ω_s_*, matches the molecular vibrational frequency. SRS does not exhibit non-resonant background and can be interpreted directly. The SRS signal occurs at the same frequency, and slightly varies in intensity, as the incident laser source. In order to detect this weak SRS signal, which is on the order of 10^−4^ to 10^−5^ of the offset due to the pump laser source, and a lock-in detection is typically used with a single photodiode [7,8,9]. However, such a method restricts the SRS from performing multi-point parallel measurement, which relaxes damages to a biological specimen. An alternative method of utilizing a fast CMOS array has been demonstrated [10] but with slow modulation rate.

Here, we present an approach with CMOS-based SRS lock-in pixels in order to address the limitation of currently available technology. Instead of adopting a separate photodiode and lock-in amplifier, we implemented both components in each pixel to extract the weak SRS signal from a huge background. The proposed pixel structure uses a photo-charge modulator based on a lateral electric field control to achieve high-speed demodulation of SRS signal components. Through a low-pass filter section, the small demodulated components are sampled with a switched-capacitor (SC) integrator and are integrated over many samples in an SC integrator to amplify the signal while eliminating the residual offset components. An SRS CMOS image sensor chip for the proof of concept is implemented and a successful operation is demonstrated.

The remainder of this paper describes the proposed image sensor and measurement results in detail. Section 2 describes the structure and operations of the SRS pixel. Section 3 explains the 1/f noise reduction technique utilized in the lock-in amplifier. The implementation and measurement results are shown in Section 4, followed by conclusions in Section 5.

## 2. Demodulator and Readout Circuits in SRS Lock-in Pixels

### 2.1. SRS Signal and Lock-in Pixel

**Figure 1:**
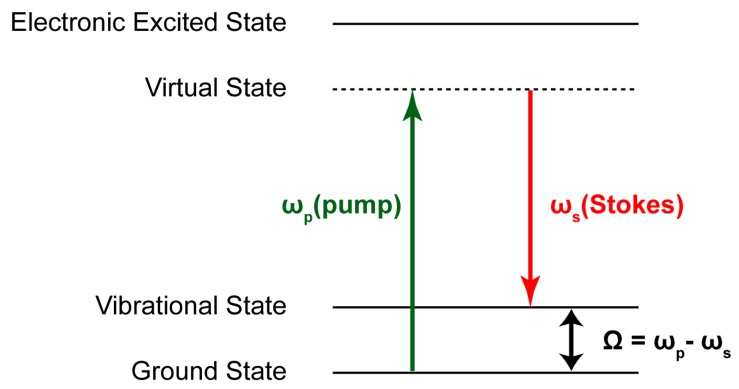
Energy bands in stimulated Raman scattering (SRS).

**Figure 2:**
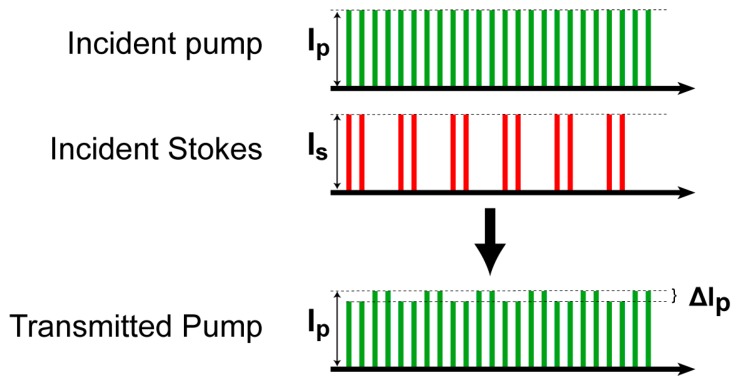
High-speed modulation for small SRS signal.

The principle of SRS is illustrated as an energy diagram in Figure 1. If the frequency difference between pump and Stokes pulses matches a particular molecular vibrational frequency, and energy transfer happens between the two laser pulses, where picosecond laser pulses with high peak power are usually used for SRS because SRS is nonlinear optical phenomena. The Stokes beam is modulated, and, consequently, the intensity of the resulting pump is varied, as shown in Figure 2. The SRS signal, which is determined by the ratio of the intensity variation (ΔI_p_) to the intensity of pump (I_p_), ΔI_p_/ I_p_ is very weak (<10^−4^). Detection of such a small signal is a big challenge because photon shot noise of the pump laser is typically greater than the interested SRS signal if the observation time is not enough and the intensity of the lasers fluctuates. Despite that, high frequency (>1 MHz) of modulation and detection with sufficient observation time can effectively reduce the effect of the laser noise, such as 1/f noise and laser fluctuation, which generally appears at low frequencies [5,11].

**Figure 3:**
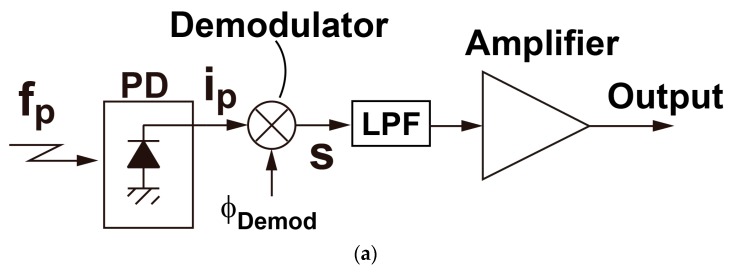

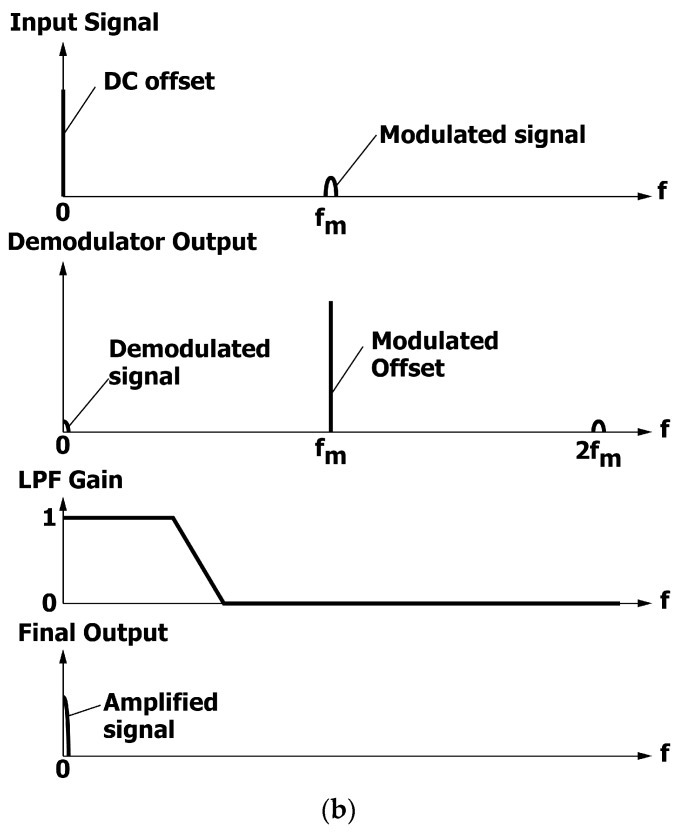
Lock-in amplifier for detecting small signal with large offset: (**a**) block diagram; (**b**) operation in frequency domain.

A general configuration for a lock-in amplifier for detecting a small AC modulated signal in a large offset is shown in Figure 3. The AC modulated signal at around frequency f_m_ is demodulated and converted to a signal at around DC and the DC offset of the input is modulated and converted to an AC component at f_m_ by a demodulator. A small DC signal component only appears at a low-pass filter (LPF) output if the LPF can suppress the AC component of the demodulator output. The small DC signal component is integrated over multiple samples, which is essentially an averaging, to reduce the photon shot noise and the other noises that may be superimposed in it due to the succeeding circuits and systems.

### 2.2. Lock-in Pixel Design for AC Signal Detection in Large DC Offset

**Figure 4:**
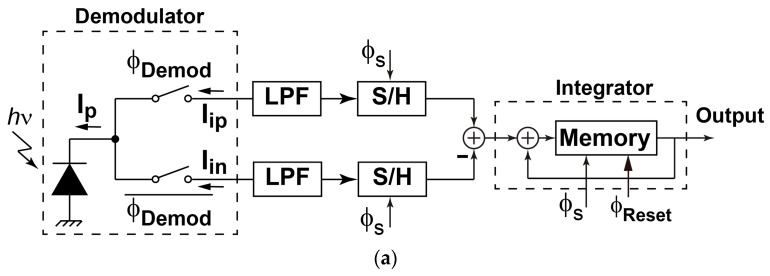

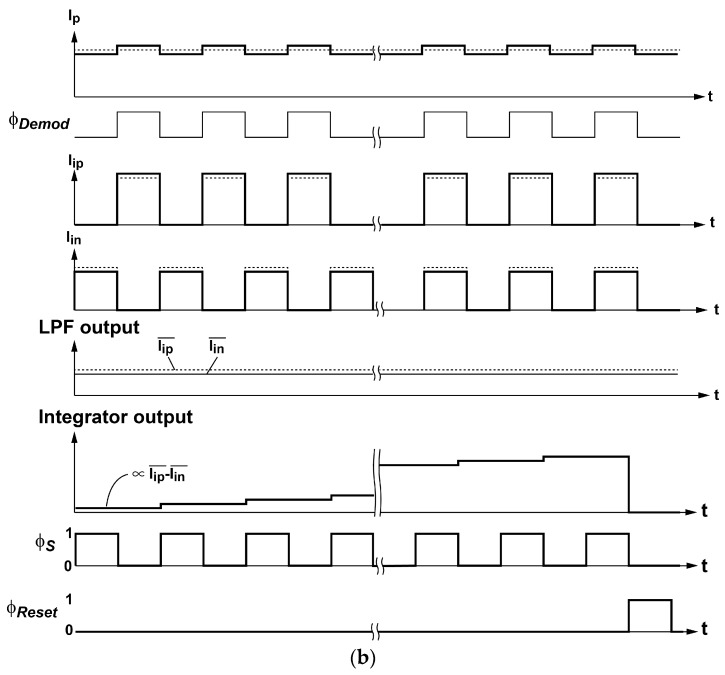
Lock-in Pixel Using a Two-Tap Demodulator: (**a**) block diagram (**b**) operation of the lock-in pixel.

A conceptual circuit diagram for implementing the lock-in pixel for a small AC signal detection in a large DC offset (unmodulated pulse train) is shown in Figure 4a. The operation of the lock-in pixel if the input signal is a pulse-modulated AC signal with continuous DC offset is shown in Figure 4b. The function of the demodulator, which converts the AC modulated signal into a signal at around DC, is expressed as:

(1) $$\{\begin{array}{c} I_{ip}=I_{p},\;I_{in}=0\;\left(\emptyset_{Demod}=\prime \prime 1\prime \prime \right)\\ I_{ip}=0,\;I_{in}=I_{p}\;\left(\emptyset_{Demod}=\prime \prime 0\prime \prime \right) \end{array}$$

where *I_p_* is the photo current of the photo detector, *I_ip_* and *I_in_* are output currents of the two-tap demodulator, and φ*_Demod_* is the demodulation clock. If the cutoff frequency of the LPF is much smaller than the modulation clock frequency, the LPF outputs contain DC components consisting of the DC offset and the demodulated signal. The difference of the two outputs contains the demodulated signal only and is amplified by the amplifier section.

### 2.3. Demodulator Design

**Figure 5:**
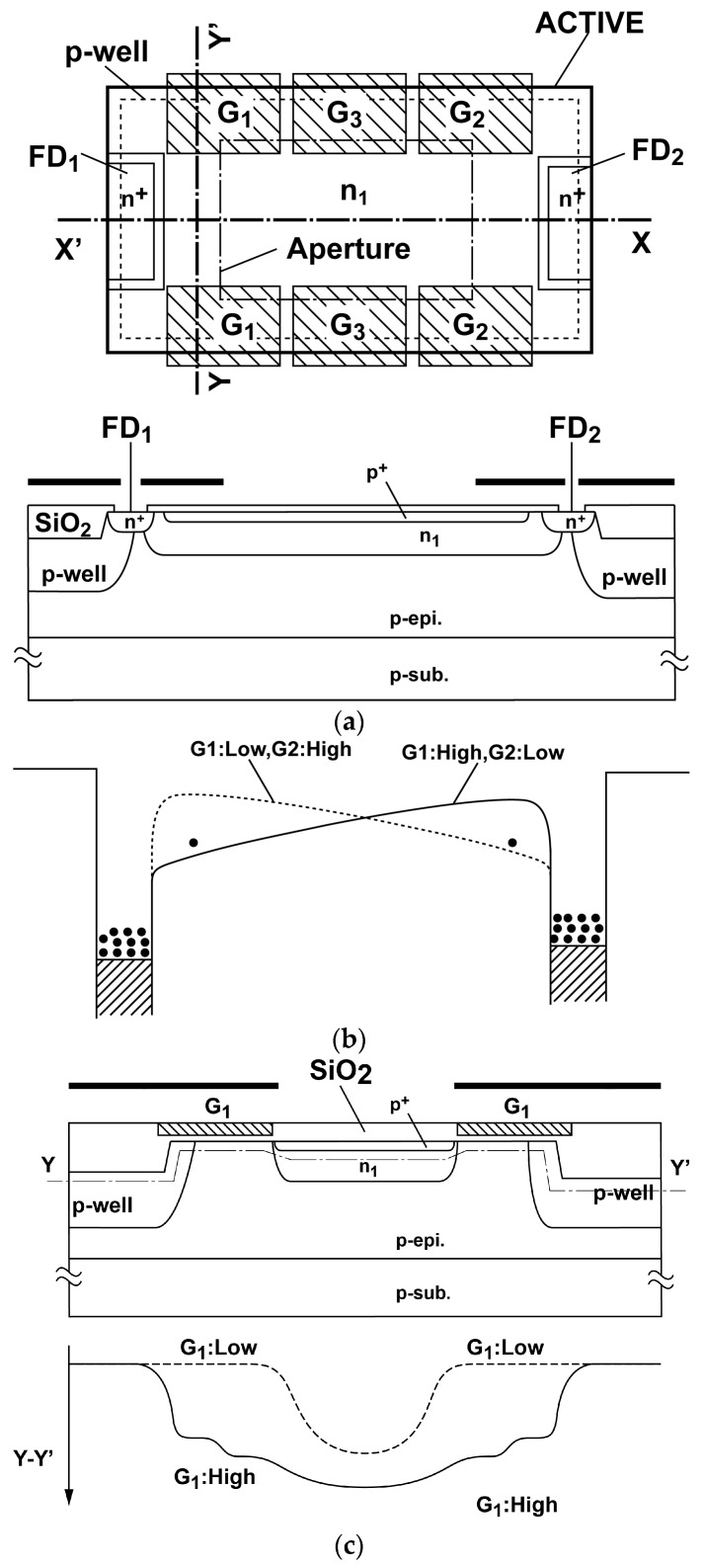
Lateral Electric Field Charge Modulator (LEFM): (**a**) top view and cross-sectional view for X-X’ (**b**) potential profiles for photo-electrons transfer (**c**) cross-sectional view and potential profile for Y-Y’.

The high-speed demodulator, which is expressed as a set of two switches in Figure 4a can be implemented with gated photo-detectors [11]. To implement the high-speed demodulator using a standard CMOS image sensor technology with a pinned photodiode option, the authors have proposed a new type of photo-charge modulators, a so-called lateral electric field modulator (LEFM) [12]. Figure 5 shows the top view of a two-tap LEFM used in the lock-in pixel for SRS signal detection. The two-tap LEFM has a pinned photodiode with a set of three gates G_1_, G_2_ and G_3_ to apply and control the lateral electric field of X-X′ direction in the pinned photodiode by the modulation of its depleted potential. In the central gate G_3_, a middle constant voltage (e.g., 0 V) is applied. Small positive voltage (e.g., 2 V) or negative voltage (e.g., −1.3 V) are used for G_1_ and G_2_ to control the lateral electric field of X-X’ direction. As shown in Figure 5b, the application of high (H) and low (L) voltage levels to G_1_ and G_2_, respectively, creates potential profiles for transferring photo electrons to the left and application of L and H to G_1_ and G_2_, respectively, creates the potential profiles for transferring to the right. The mechanism of the modulation of depleted potential in the pinned photodiode can be explained by Figure 5c which shows the cross-section and potential profile of Y-Y’ direction. For instance, by applying a low voltage (e.g., −1.3 V) in the gate G_1_, the potential under the gates is lowered to around 0 V and a resulting voltage of the pinned diode is set to relatively low voltage. When a relatively high voltage (e.g., 2 V) is applied to the gates, the potential under the gate and the resulting potential in the pinned diode are raised to high because of the modulation of fringing electric field due to the applied gate voltage change of the set of gates G_1_. The LEFM ensures a very high-speed electron transfer because this structure does not have a problem of creation of potential barrier at the edge of the transfer gate and charge trapping under the gate (Si-SiO_2_ interface) unlike conventional transfer technique using a transfer gate in the signal path.

**Figure 6:**
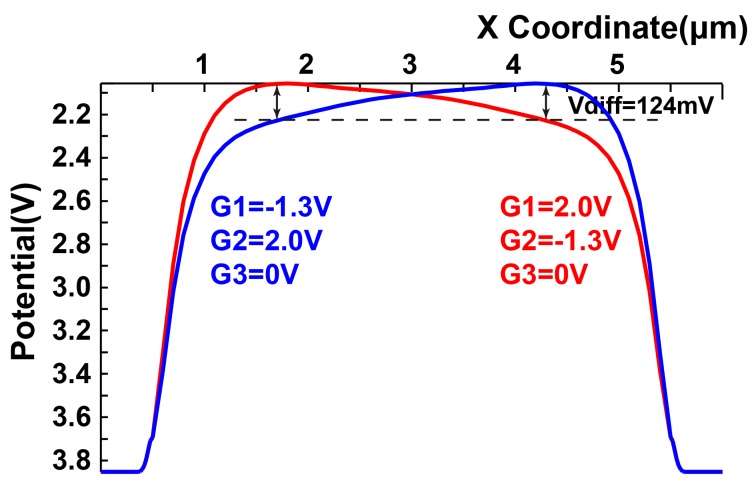
Simulation result of potential profile along X coordinate.

In order to investigate the characteristics of the used LEFM detector shown in Figure 5, a simulation has been conducted using a device simulator SPECTRA. The implementation using 0.11 µm CMOS image sensor (CIS) technology with a lightly-doped substrate is assumed, and device parameters based on this technology are used for the device simulation. A simulation result of the potential profile along the transfer channel in the LEFM is shown in Figure 6. In this simulation, the potential profiles for the cases G_1_ and G_2_ are set to −1.3 V and 2 V, respectively, and G_1_ and G_2_ are set to 2 V and −1.3 V, respectively, are shown. The gate voltage of G_3_ is set to always 0 V. By using a 3.3 V (−1.3 V to 2 V) for gate driving voltage difference between G_1_ and G_2_, a maximum potential modulation of approximately 124 mV is created for the distance of 2.6 µm resulting in a high electric field along the X coordinate. This electric field allows us to have a photo-electron transfer time of less than one nanosecond and is sufficient for the demodulation clock of 20 MHz in this design.

**Figure 7:**
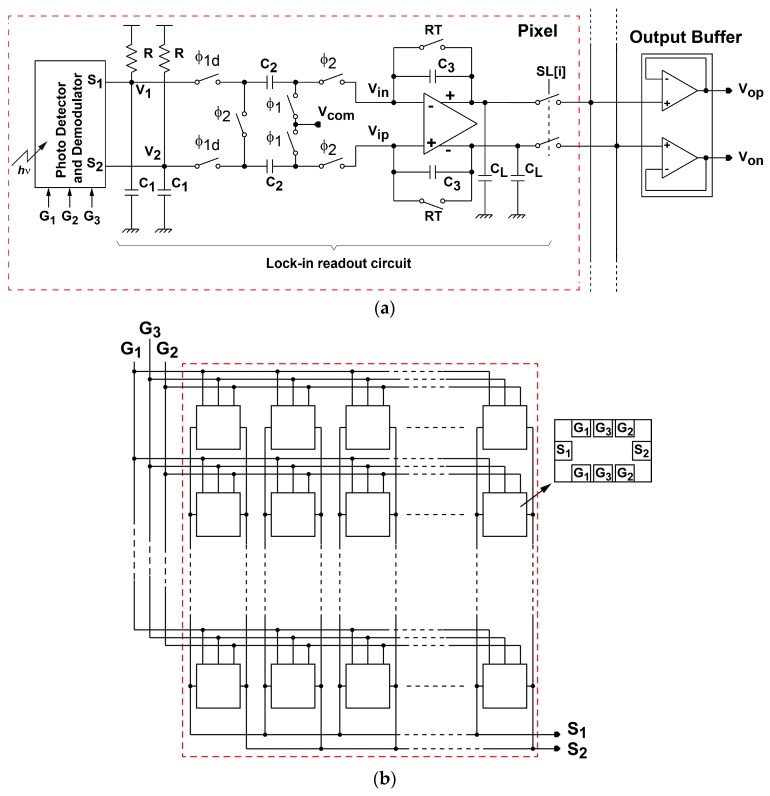
Lock-in pixel readout circuit: (**a**) lock-in readout circuit (**b**) photo detector and demodulator.

In the actual pixel design, a 10 × 10 array of the LEFM units where all the corresponding outputs are connected in parallel is used in each pixel as shown in Figure 7b.

### 2.4. Pixel Readout Circuit Design and Operation

**Figure 8:**
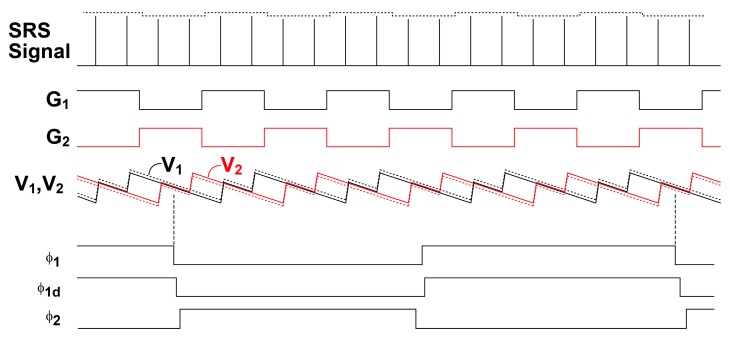
Timing diagram for the lock-in pixel operations.

**Figure 9:**
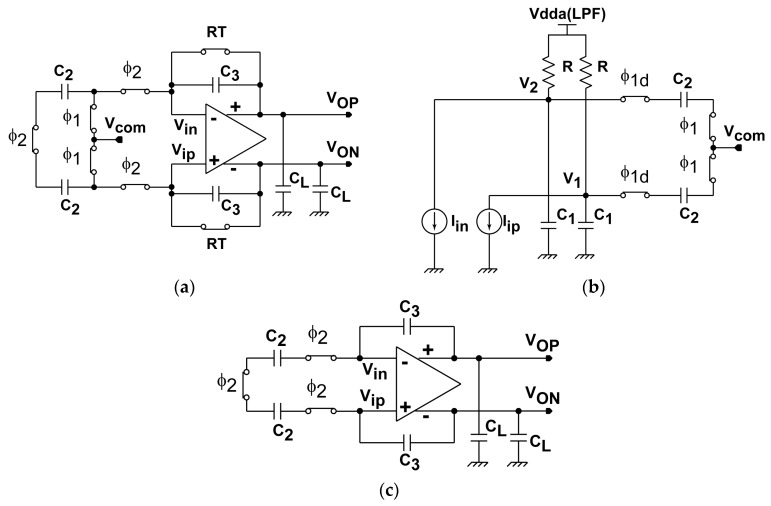
Phase diagrams of the lock-in readout circuits: (**a**) reset phase; (**b**) sampling phase (**c**) transfer phase.

**Figure 10:**
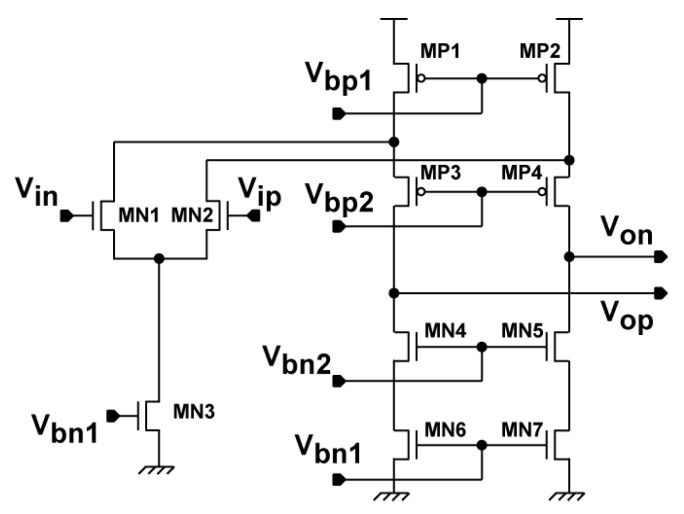
Schematic diagram of the fully differential folded-cascode operational amplifier.

The readout circuit together with the photo detector and demodulator for the SRS lock-in pixel imager is depicted as shown in Figure 7. The LPF for extracting the demodulated SRS signal is implemented with a 1st-order RC filter. The sampling of the LPF outputs and discrete-time integration of the difference of the sampled LPF output signals are implemented with a fully-differential switched-capacitor (SC) integrator. The timing chart for the operation of the lock-in pixel readout circuits and waveform of the LPF outputs are shown in Figure 8. The timing G_1_ and G_2_, which controls the demodulation of the two-tap LEFM, are appropriately set to sample the DC signal exactly during the Stokes on and Stokes off, respectively The phase diagram of the readout circuits is shown in Figure 9. Initially the integrator is reset by turning on the switches controlled by RT, φ_1_, and φ_2_ as shown in Figure 9a. After the integrator is reset, the LPF outputs are amplified by the SC integrator by two steps. First the LPF outputs are sampled by the equivalent circuit shown in Figure 9b when switches controlled by φ_1_ and φ_1d_ are turned on, and then the sampled differential charges stored in two capacitors of C_2_ in the sampling phase are transferred to C_3_ by using the equivalent circuit shown in Figure 9c when the switches controlled by φ_2_ are turned on. The operation with the sampling (Figure 9b) and charge transfer (Figure 9c) gives one cycle of the discrete-time integration, and the gain of the SC integrator is given by the number of cycles. A fully-differential folded-cascode operational amplifier is used in the readout circuit, as shown in Figure 10. V_in_ and V_ip_ are the differential inputs, V_on_ and V_op_ are the differential outputs, while V_bn_ and V_bp_ are the biasing voltages for the operational amplifier. The phase margin of the operational amplifier is 79 degrees.

To pick up and amplify the small demodulated signal while suppressing the large modulated DC offset, the sampling timing is very important. To find the best timing to minimize the offset, the detailed waveform after the demodulation and low-pass filtering must be considered. The actual offset signal, which is called DC offset here, is a pulse train whose amplitude is unmodulated. The waveform after the low-pass filter becomes a solid-line of V_1_ and V_2_ is shown in Figure 8. If the input light pulse train is modulated by an AC signal, the waveform becomes the dashed-line of V_1_ and V_2_ in Figure 8. By choosing the timing of sampling pulses, φ_1_ and φ_1d_ as shown in Figure 8, it is understood that the residual DC offset is minimized and the demodulated AC signal is detected and amplified by the integrator. With the capability of extracting the SRS signal in the pixel, parallel detection of the SRS signal in a line sensor for an SRS spectrum or an area sensor for an SRS image is feasible.

## 3. 1/f Noise Reduction of a Lock-in Amplifier

**Figure 11:**
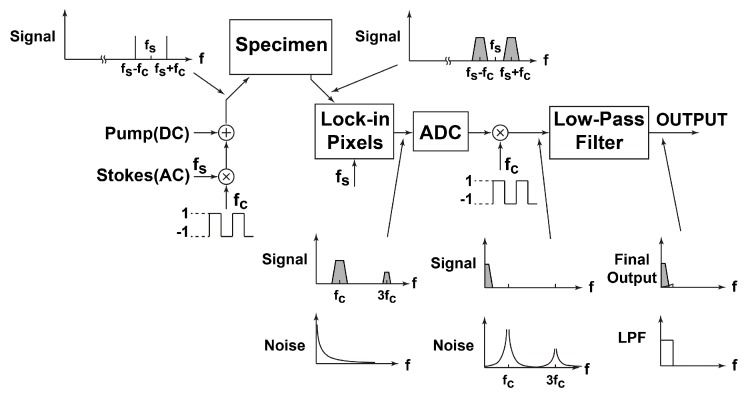
Double modulation technique to reduce low-frequency noise component and residual offset component.

Residual offset and low-frequency noise (1/f noise) are reduced in two steps using the double modulation technique where the principle is depicted in Figure 11. For further detailed description of the double modulation technique, the fact that the laser light is actually a high-frequency pulse train whose frequency is four times the modulation frequency should be considered in the frequency spectrum, but such high-frequency components are omitted in Figure 11 for simplicity. The LPF in the lock-in pixel is not sufficient to fully attenuate the DC offset. Therefore, the phase delay between the demodulated pixel output signal and the readout sampling signals φ*_1_,* φ*_1d_* and φ*_2_* should be carefully adjusted to ensure that the offset signal is canceled out or reduced to the minimum in order to achieve a high dynamic range. If there is no light or just a DC light, in fact, light pulses are used but the amplitude of pulses is unmodulated, is applied to the sensor, and the amplifier differential output should be reduced to be as small as possible by a careful choice of the phase delay value. The double modulation technique is then used to reduce the low-frequency noise component and the residual offset component, which are also introduced by the lock-in pixel circuitry. Initially, AC light signal (SRS signal) with the modulation frequency of *f_s_* is multiplied by the square wave signal of frequency *f_c_* through a function generator in order to change its polarity at every half cycle of the square wave. After this first modulation, the AC light signal has mainly two frequency components *f_s_* − *f_c_* and *f_s_ + f_c_* and their harmonics. The DC light signal (unmodulated pump laser pulses) and AC light (modulated Stokes laser pulses) irradiate the specimen and the output from the specimen, the modulated pump laser, is received by the lock-in pixels. The SRS signal due to molecular vibration is observed from the specimen, and it results in the modulation at the frequency components *f_s_* − *f_c_* and *f_s_ + f_c_*. The lock-in pixel, which is locked at the frequency f_s_, demodulates and amplifies the SRS signal at the pixel output. The SRS signal modulated at frequencies f_c_ and its odd harmonics are produced. The pixel output is converted to a digital number using an A-to-D converter in the external system. The demodulation of the modulated SRS signal at the frequency *f_c_* is done in the digital domain by multiplying a square wave with the frequency of *f_c_* and that takes 1 or −1, and then applying a digital low-pass filter. This demodulation process shifts the low-frequency noise components due to the 1/f noise of the lock-in amplifier and residual offset to a higher frequency around *f_c_* and its harmonics, and the shifted noise and residual offset are suppressed by applying the digital low-pass filter. Although white noise increases when 1/f noise is reduced, white noise can then be reduced by averaging and increasing the time of observation. The same cannot be done for 1/f noise.

## 4. Implementation and Measurement Results

### 4.1. Implementation of CMOS SRS Pixel Array Chip

**Figure 12:**
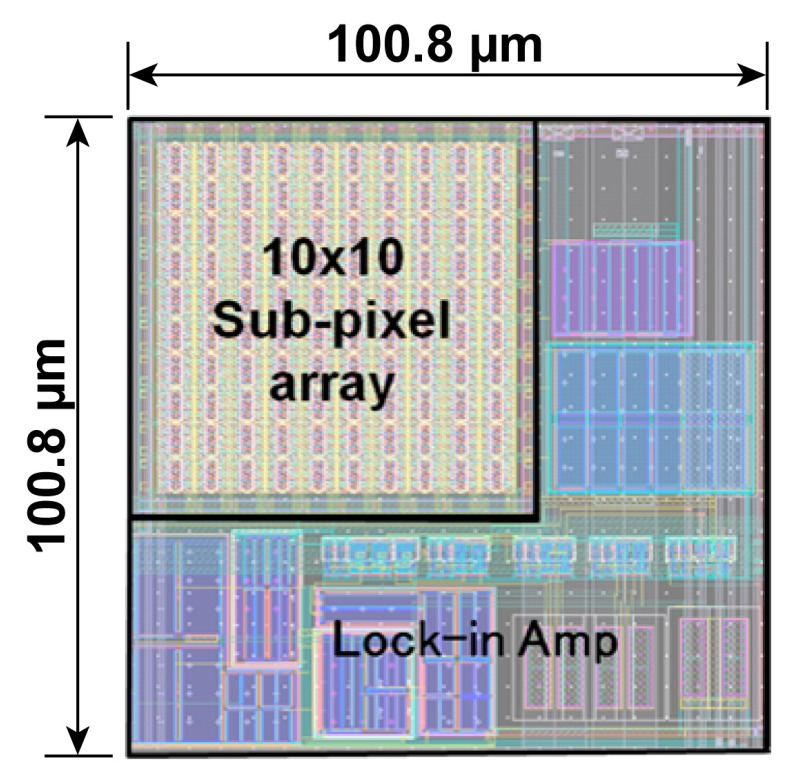
SRS pixel layout showing the sub-pixel array and lock-in amplifier.

An SRS CMOS imager chip has been fabricated using 0.11 µm CMOS image sensor process technology to experimentally evaluate the performances of the proposed circuit. Figure 12 shows the layout of the SRS CMOS pixel. A photo receiver with 10 × 10 sub-array of demodulators and lock-in readout circuits are put into the pixel area of 100.8 µm × 100.8 µm. The entire chip consists of an array of 64 × 8 pixels, a pixel selector and buffer amplifiers. As a preliminary study for the operation as an SRS imager, only a single pixel is characterized.

### 4.2. Experimental Setup and Measurement Conditions

**Figure 13:**
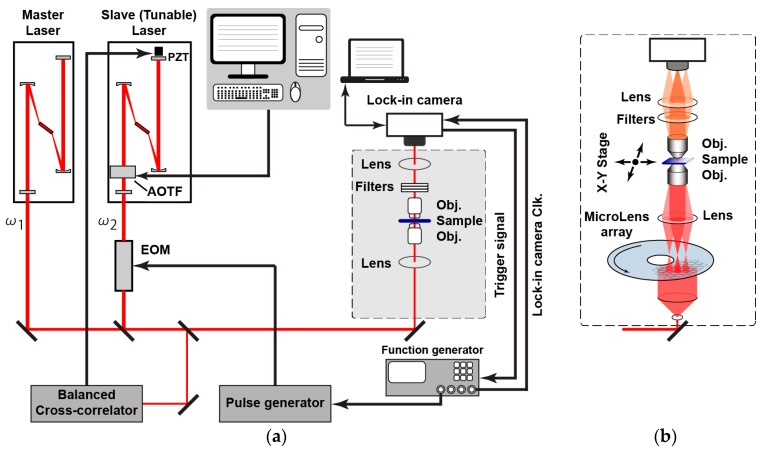
Experimental setup for SRS: (**a**) the setup used to characterize the pixel; (**b**) the lens setup to be used for image sensor array.

**Figure 14:**
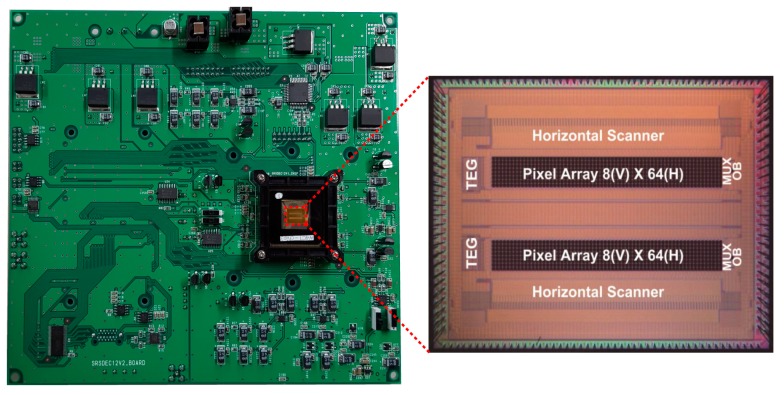
Photograph of the PCB sensor board with micrograph of the prototype SRS CMOS imager. The chip consists of 64 × 8 pixel array, horizontal scanner as pixel selector, and output buffer amplifiers.

**Figure 15:**
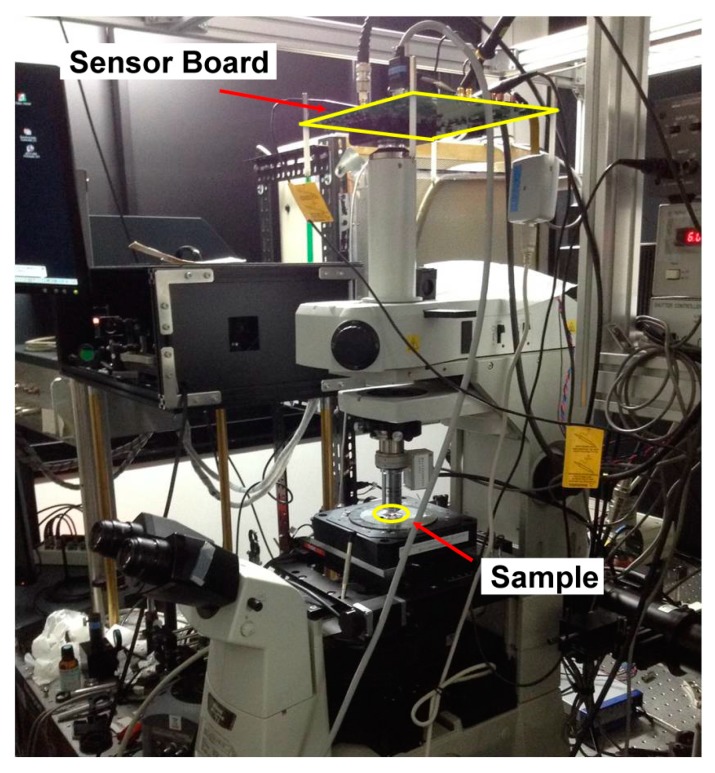
Photograph of the measurement setup for SRS.

Figure 13a shows the simplified illustration of the experimental setup to characterize the lock-in pixel of the proposed SRS imager. Two laser sources are used; one is the master laser, also called a pump laser to generate the unmodulated (DC) laser light running at 80 MHz with a fixed wavelength of 709.5 nm, and the other is the tunable slave laser, also called a Stokes laser to generate the modulated (AC) laser light running at 20 MHz where the wavelength is initially fixed at 837 nm. The Stokes laser wavelength can be tuned using the computer controlled acousto-optic tunable filters (AOTF) for SRS spectrum measurement requirements. The proposed imager is incorporated to the lock-in camera controlled by a computer and being synchronized to the function generator. The other channel of the function generator is triggered by the lock-in camera to change the polarity at each frame in order to achieve the double modulation technique. The experimental setup includes also a balanced cross-correlator for synchronization of pump and Stokes lasers and an electro-optic modulator (EOM) for modulating Stokes laser beam [13]. Figure 13b shows the lens setup for further works of an array of pixels to be used to obtain the image, with other experimental setups being identical. The microlens array [14] splits laser beams into multiple collimated beamlets to be focused on multiple spots of the specimen. The multi-points SRS signal will then be detected in parallel with the proposed SRS imager. Besides the use of an array of pixels and parallel detection, *x*-*y* stage scanning will also be utilized to obtain a high-definition image. If a large density detector is accomplished, high-speed imaging could be realized by rotating the microlens array disk for scanning the foci [14]. The printed circuit board (PCB) sensor board and chip micrograph are shown in Figure 14. Figure 15 shows the photograph of the real measurement setup for SRS with the developed CMOS imager sensor board mounted on top of the microscope. 

The measurement conditions for the following results are shown in Table 1. The pump and Stokes laser powers are 3.8 mW and 8.0 mW, respectively. Pump laser wavelength is fixed at 709.5 nm while Stokes laser wavelength is swept from 837 nm to 846 nm with step size of 0.25 nm. The sampling at the frequency of 5 MHz during the integration time of 150 µs yields a gain of 750. The sensitivity of the pixel for quantum efficiency of 40% at 800 nm is 0.258 A/W. realized

### 4.3. Measurement Results

For SRS measurement, the pump beam is separated from the modulated Stokes beam using optical filters, and the modulation appearing on pump beam is detected. Here, for evaluation of the lock-in pixel performance, DC pump laser pulses and the AC Stokes laser pulses are input into the pixel to stimulate the SRS signal.

**Figure 16:**
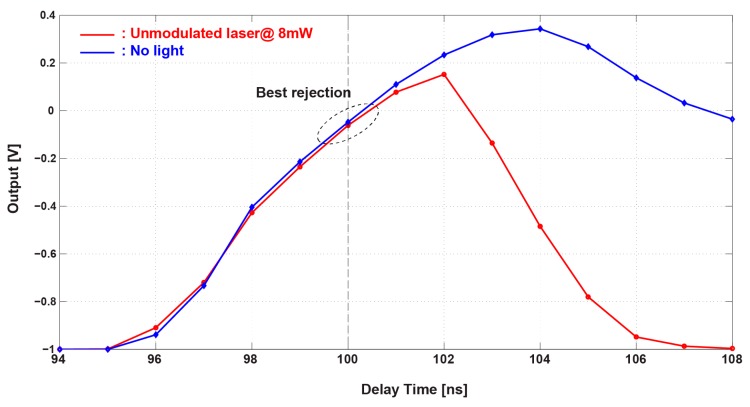
Delay time adjustment to minimize the residual offset in the pixel output.

As described in Section 2.4, the phase delay between the demodulator clock and the readout sampling clocks φ*_1_,* φ*_1d_* and φ*_2_* must be carefully tuned to minimize the residual offset in the pixel output. The optimal phase can be found where the amplifier differential output when a DC laser is applied has approximately the same value when no light input is applied. Figure 16 shows the measurement results of the pixel outputs for a DC light and no light input as a function of the common phase delay of sampling clocks φ*_1_,* φ*_1d_* and φ*_2_*. Because of the coupling of gating clock noise for the demodulator to analog circuits of the lock-in pixel, the output with no light also has a large variation to time. The best phase delay to meet the abovementioned condition is around 100 ns. The following measurement results use the delay of 100 ns.

**Figure 17:**
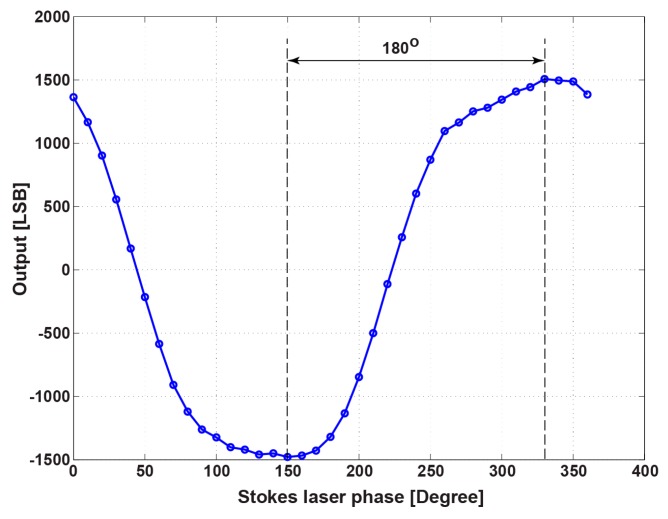
Pixel output as a function of the laser modulation.

To maximize the demodulation sensitivity to the SRS signal, the phase of the demodulator clock to the AC laser light must be adjusted. Figure 17 shows the lock-in pixel output as a function of Stokes laser phase change. The differential output after A/D conversion with the unit of least significant bit (LSB) is shown. Maximum output values are obtained at 150° and 330°. This shows that there is a phase difference of 180° between the two maximum values of the differential output signal. In the following measurements, the phase of the AC-modulated laser is set at 330° to maximize the sensitivity.

**Figure 18:**
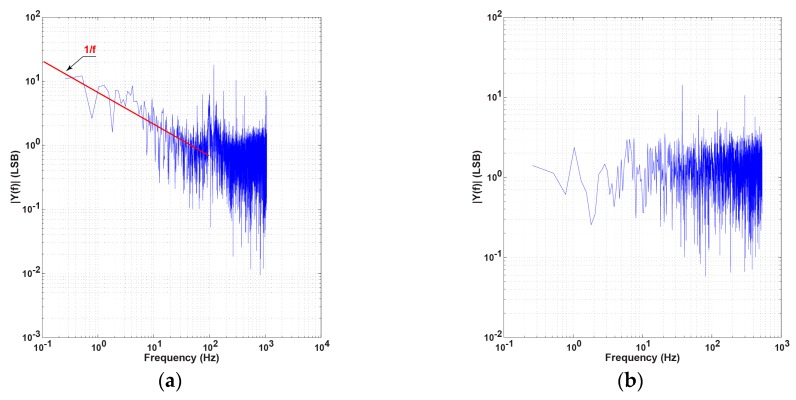
1/f noise reduction effect of the double modulation: (**a**) without double modulation technique (**b**) with double modulation technique.

In order to demonstrate the effectiveness of the double modulation technique to reduce the residual offset signal and low frequency noise, two different noise measurements were done, with and without the double modulation technique. Figure 18 shows the measured noise spectrum of the pixel output with and without the double modulation technique. The spectrum is obtained by the 8192 points fast Fourier transform (FFT) to the measured time-domain sequence of the pixel output. Only an unmodulated laser signal is applied. Using the double modulation technique as shown in Figure 18b when compared with Figure 18a, in which the double modulation is not used, the low-frequency noise component, particularly 1/f noise, is suppressed effectively.

**Figure 19:**
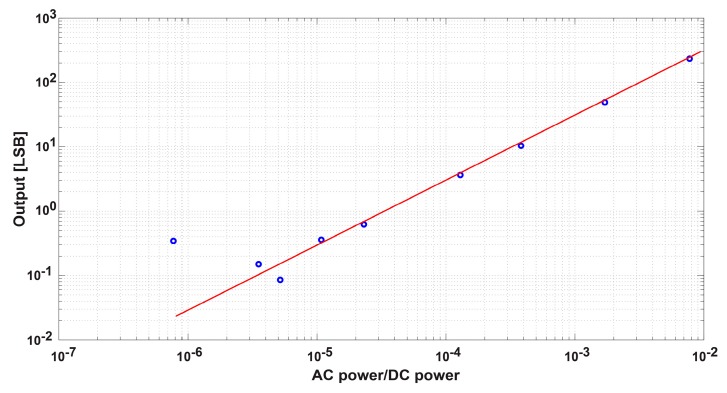
Small signal detection linearity.

To show the effectiveness of the proposed circuit and the possibility of detecting a very small SRS effective signal included in a large offset component, the linearity of the pixel output to the AC signal power normalized to the DC power is measured as shown in Figure 19. In this measurement, the power of the DC laser is kept unchanged while the measurements are done by changing AC laser power only. A plot of the averaged values of six measurements is shown. The time taken for each measurement is about five minutes, which includes the manual changing of the filter. A good linearity for a power ratio from 10^−2^ to 10^−5^ is obtained. In the region of very small AC power (<10^−5^), the output signal is not stable. One of the reasons is that, in the range of <10^−5^, the output signal from the pixels becomes very small, and, if it is smaller than 1 LSB, the signal after A/D conversion is much influenced by the nonlinearity of the A/D converter used.

**Figure 20:**
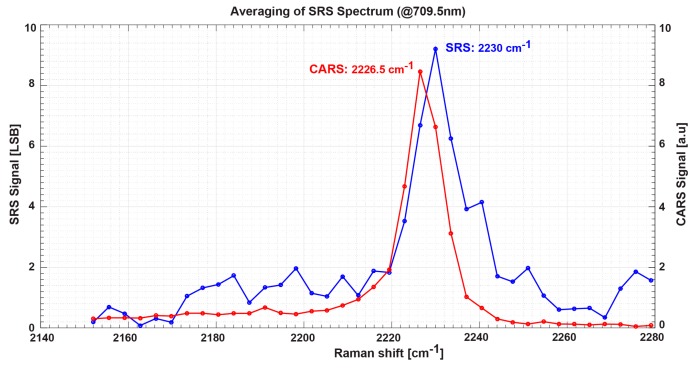
Benzonitrile Raman spectrum measured by coherent anti-stokes Raman spectroscopy (CARS) (**red**) and SRS (**blue**).

A sample of Benzonitrile prepared on a glass sample holder is used to confirm the effectiveness of the proposed lock-in pixel for SRS spectrum measurements. The measured SRS spectrum is shown in Figure 20. The measurement is currently performed manually with measurement time of about 15 min. Automated measurement will be performed in future work where a trigger signal is sent from the sensor board at regular intervals to the laser system for wavelength tuning and capture. The figure also shows the spectrum simultaneously measured using coherent anti-stokes Raman spectroscopy (CARS) for comparison purposes. According to the standard Raman spectra [15], Benzonitrile should have a Raman shift peak at 2229.4 cm^−1^. Measured SRS spectrum using the SRS lock-in pixel shows a clear Raman shift peak at 2230 cm^−1^. In the spectrum measured by CARS, the Raman shift peak appears at 2226.5 cm^−1^. These differences are might be due to the distortion of CARS spectrum by the effect of the non-resonant background. The noises that appear in the SRS spectrum are partly due to the limitation of the A/D converter resolution as discussed earlier in this section. A higher resolution A/D converter will be used in future work to improve the linearity. Improvement of signal-to-noise ratio can be further enhanced by modification of both detector and circuit. The use of the array of sub-pixels to achieve a large area detector in this imager might have deteriorated the signal-to-noise ratio. A large area LEFM should improve the stability of the output and thus improve signal-to-noise ratio. Additionally, a higher analog gain of the lock-in amplifier will be implemented to further boost the signal-to-noise ratio. Although the Raman shift obtained by the SRS lock-in pixel is noisy, the possibility of Raman shift spectra measurement using the CMOS lock-in pixel is recognized by Figure 20.

## 5. Conclusions

In this paper, a high-speed lock-in pixel technology for stimulated Raman scattering CMOS imagers is presented. The proposed lock-in pixel with a high-speed demodulator using a lateral electric field control of charge and the double modulation technique for reducing residual offset and low frequency noise components successfully detects small stimulated Raman scattering signals in huge offset due to the direct pump laser light. Careful setting of the phase delay between the LEFM demodulated output signal and the readout sampling clock suppresses the residual offset and prevents the saturation of the amplifier output by the offset when a large gain of the SC integrator is used. However, we acknowledge that it is challenging to implement the phase delay across an array of pixels. The implemented lock-in pixel successfully detects a small AC signal whose AC/DC power ratio is less than 10*^−^*^5^. The SRS spectrum of Benzonitrile has been successfully measured. These results are a significant advancement toward the goal of realizing the SRS CMOS image sensor.

## Figures and Tables

**Table 1:** Measurement conditions.

Parameter	Value
AC Power	3.8 mW
DC Power	8.0 mW
Pump laser wavelength	709.5 nm
Stokes laser wavelength	837~846 nm
Step size	0.25 nm
Modulation frequency	20 MHz
Integrator sampling frequency	5 MHz
Integration time	150 µs
Number of integration (Gain)	750
